# Impact of Engineered Zinc Oxide Nanoparticles on the Individual Performance of *Mytilus galloprovincialis*

**DOI:** 10.1371/journal.pone.0061800

**Published:** 2013-04-17

**Authors:** Shannon K. Hanna, Robert J. Miller, Erik B. Muller, Roger M. Nisbet, Hunter S. Lenihan

**Affiliations:** 1 Bren School of Environmental Science and Management, University of California Santa Barbara, Santa Barbara, California, United States of America; 2 Marine Science Institute, University of California Santa Barbara, Santa Barbara, California, United States of America; 3 Department of Ecology, Evolution and Marine Biology, University of California Santa Barbara, Santa Barbara, California, United States of America; University of California, Merced, United States of America

## Abstract

The increased use of engineered nanoparticles (ENPs) in consumer products raises the concern of environmental release and subsequent impacts in natural communities. We tested for physiological and demographic impacts of ZnO, a prevalent metal oxide ENP, on the mussel *Mytilus galloprovincialis*. We exposed mussels of two size classes, <4.5 and ≥4.5 cm shell length, to 0.1–2 mg l^−1^ ZnO ENPs in seawater for 12 wk, and measured the effect on mussel respiration, accumulation of Zn, growth, and survival. After 12 wk of exposure to ZnO ENPs, respiration rates of mussels increased with ZnO concentration. Mussels had up to three fold more Zn in tissues than control groups after 12 wk of exposure, but patterns of Zn accumulation varied with mussel size and Zn concentrations. Small mussels accumulated Zn 10 times faster than large mussels at 0.5 mg l^−1^, while large mussels accumulated Zn four times faster than small mussels at 2 mg l^−1^. Mussels exposed to 2 mg l^−1^ ZnO grew 40% less than mussels in our control group for both size classes. Survival significantly decreased only in groups exposed to the highest ZnO concentration (2 mg l^−1^) and was lower for small mussels than large. Our results indicate that ZnO ENPs are toxic to mussels but at levels unlikely to be reached in natural marine waters.

## Introduction

Increasing production of engineered nanoparticles (ENPs) for use in a variety of applications has raised concern about their potential ecological impacts [1], [2]. The increased reactivity and other special properties associated with the small size of ENPs make them ideal for application in a variety of consumer products, such as cosmetics, electronics, clothing, and tires, and as manufacturing aids in the form of catalysts, conductors, and semi-conductors [1], [3]. As a result, the rate at which ENPs are being released into the environment is increasing [4]. Yet there is a growing body of evidence demonstrating that ENPs are toxic and may pose ecological risks. For example, metal and metal oxide ENPs reduce the growth rates of bacteria [5], freshwater algae [6], and marine phytoplankton [7], [8], and survival of fish [9], [10] and crustaceans [11]. While these studies show that ENPs are potentially hazardous, many of them used short-term laboratory exposure tests with relatively short-lived species, which hinders the use of the results in making ecologically relevant predictions. A compounding problem is our inability to measure accurately the typically low ENP concentrations in the environment. Despite those uncertainties, environmental loading models [12] suggest that most ENPs are not substantial environmental risk factors at present.

An important role of ecotoxicology is to assess the hazard potential of anthropogenic contaminants before they become real environmental problems. One way forward is to test the influence of ENPs on ecologically-important organisms, such as keystone or ecological engineer species [13] in life-cycle experiments. However, many such species are long-lived, so it is only feasible to determine the impact of ENPs on the performance of individuals during relatively small portions of their life histories, and then use the information obtained with those studies to model the potential impact over entire life spans of the animals, with the caveat that different life stages may have different susceptibility and therefore responses to the same type and level of contaminant [14]. To better understand the potential impacts of ENPs on populations of long-lived organisms, we can couple laboratory-based studies with modeling, thereby allowing us to simulate the effects of ENPs on populations based on the results of individuals.

Here we test whether ZnO ENPs impact the physiological performance and survival of the marine mussel, *Mytilus galloprovincialis*, a coastal marine ecosystem engineer that helps maintain water quality through filter-feeding, builds biogenic reefs that support biodiversity, and is an important prey species to many coastal marine predators [15], [16]. ZnO ENPs are present in a number of cosmetics, sunscreens, and coatings [1], [17] and therefore, may potentially be released into marine environments in biologically meaningful quantities. While the effects of ZnO ENPs on mussels are unknown, Zn has been shown to decrease enzyme activity in mussel tissue [18], [19] and increase metabolism during recovery in clean water [20]. Mussels accumulate Zn when exposed to particulate or dissolved forms [21], and concentrate Zn especially in cells along the gut and in the mantle and gills [22], with smaller animals having higher body burdens of Zn compared to larger ones [23]. Zn tissue concentration is negatively correlated with growth rate in mussels [24] and survival decreases with increasing environmental Zn concentrations [25]. Based on what is known about Zn toxicity to mussels, we test the hypotheses that mussels exposed to ZnO ENPs will 1) increase respiration rate after exposure, 2) accumulate Zn throughout the experiment, 3) decrease growth and survival, and 4) show size dependence during the experiment, with smaller mussels having more pronounced effects than larger mussels. We fit descriptive regression models that describe the size and dose dependence of the various physiological and demographic rates.

## Methods

### Mussels

*Mytilus galloprovincialis* with shell lengths of approximately 2–6 cm were obtained from Taylor Shellfish Farms (Shelton, WA, USA). Mussels were housed in flowing, sand-filtered seawater for 1 wk prior to the start of experiments. A total of 4,400 mussels were used in ZnO ENP exposures. We separated mussels into small (<4.5 cm total shell length) and large (≥4.5 cm total shell length) size classes and divided them into 40 aerated polystyrene tanks, each containing 5 l of sand-filtered seawater. Tanks of small mussels contained 120 individuals while tanks of large mussels contained 100 individuals. Mussels were allowed to acclimate to their tanks for three days prior to experiments. We exposed small and large mussels to ZnO ENPs at 0.1, 0.5, 1, and 2 mg l^−1^ for 12 wk by adding ZnO ENPs along with feed to tanks 5 d each wk with sand-filtered seawater, animals in control tanks were given only feed mixed with sand-filtered seawater. At each water change, tanks were emptied of water, rinsed with seawater, refilled with seawater, and the appropriate amount of ENP/feed mixture was added. Dead mussels were removed if present.

### ENPs and Suspension Preparation

ZnO ENPs were obtained from Meliorum Technologies (Rochester, NY, USA) and characterized by the University of California Center for the Environmental Implications of Nanotechnology (UC CEIN) as spheroid, 100% zincite and 20–30 nm in diameter [26], [27]. We prepared stock suspensions for experiments by adding ZnO ENPs to purified water (Barnstead Nanopure, Thermo Fisher Scientific, Waltham, MA, USA, resistivity >18 MΩcm) to make a 1 g l^−1^ suspension and sonicating in a bath sonicator for 30 min. We diluted this suspension in filtered seawater (0.45 µm) containing 10 mg l^−1^ alginate to prepare a 100 mg l^−1^ suspension of ZnO ENPs. We then sonicated this suspension for 10 min, added commercially prepared phytoplankton (Shellfish Diet 1800, Reed Mariculture, Campbell, CA, USA), which includes *Isochrysis* sp., *Pavlova* sp., *Thalossiosira weissflogii*, and *Tetraselmis* sp., and used this mixture in exposure treatments. Enough phytoplankton was added to attain a final cell count of 2×10^6^ cells ml^−1^ in each tank.

### Respiration measurements

We determined respiration rates of mussels after 12 wk of exposure (i.e., at the end of the experiment) for 12 mussels from each concentration and size class. Individual mussels were placed into a respirometer containing clean, sand-filtered seawater, a water pump to ensure mixing, and an optical oxygen probe with built-in datalogger (D-Opto, Envco, NZ). The respirometer was submerged in flowing seawater to maintain temperature, sealed, and the oxygen concentration in the chamber was measured once per minute for 1 h. We then removed mussels from the chamber and measured oxygen concentration for 1 h more to account for any other biological activity in the chamber. We measured mussel length and dry weight of tissue after dissection from the shell. These mussels were then analyzed for Zn content as described below.

### Bioaccumulation of zinc

We removed five mussels from each tank every 2 wk throughout the study and froze them for Zn analysis. Frozen mussel samples were thawed, dissected and separated into somatic tissue and gonad. Samples were dried at 60°C for 72 h. Samples were weighed to the nearest 0.1 mg, digested in concentrated trace-metal-free HNO_3_ (Sigma-Aldrich Co., St. Louis, MO, USA) for 2 h at 80°C in capped glass vials and diluted to 10% acid using purified water. We then determined Zn content of tissue and gonad samples using inductively coupled plasma atomic emission spectroscopy (ICPAES, Thermo ICAP 6300, Thermo Fisher Scientific). Blank and standard solutions were run every 10 samples.

### Growth and survival

We determined individual mussel growth by marking 20 individuals in each tank and measuring the total length (TL) of the mussels' shell over the course of the study. The outer shell of each mussel was roughened with sandpaper and numbered with white paint. We measured TL of labeled mussels using digital calipers (±0.01 mm), along the longest axis of the mussel. TL was measured prior to exposure, after 6 wk, and after 12 wk of exposure. Growth rates were calculated for individual mussels and averaged among replicates. We counted mussels in each tank after 6 and 12 wk of exposure and corrected for sampling to determine survival.

### Statistical Analysis

We tested whether mussel respiration rate, Zn somatic tissue concentration, Zn gonad tissue concentration, rate of Zn accumulation in somatic tissue, and growth rate varied as a function of ZnO ENP concentration using multiple ordinary least squares (OLS) regression models. Mussel size (small or large), ZnO ENP concentration, and the interaction of these terms were factors in the analysis for all models except Zn concentration in gonad, as gonads were only analyzed from large mussels. We predicted that increased exposure to ZnO ENPs would increase respiration rate post exposure, increase Zn loading in tissues as well as the rate at which Zn was accumulated, and decrease growth of mussels throughout the experiment. We also predicted that larger mussels would be able to tolerate higher concentrations of ZnO ENPs than smaller mussels, thus reducing the impacts of these ENPs on physiological rates. To test these predictions we constructed multiple regression models for each physiological parameter as follows:
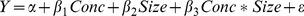
where 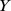 is the physiological parameter of interest (respiration rate, Zn tissue concentration, rate of Zn accumulation, or growth), *α* is the physiological parameter value of the control group of large mussels, *Conc* is the ZnO ENP concentration, *Size* indicates whether the mussel was small or large, and *ε* is the error not explained by the model. The coefficients, *β*, are estimates of the impact of the independent variable on the dependent 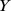. We removed the interaction term if it was not statistically significant and reran the model. When the interaction term was statistically significant, we ran separate linear regression models for small and large mussels to determine the relationship between the physiological parameter and nominal ZnO ENP concentrations. We square-root transformed ZnO ENP concentration for the respiration rate model because the response variable, respiration rate, was a non-linear function of ZnO ENP concentration. We also compared the tissue dry weight to shell TL ratio from mussels at the beginning of the experiment with that of mussels at the end of the experiment to determine if mussels gained or lost weight during the study. This analysis was conducted using OLS regression similar to the above, with the tissue dry weight to shell TL ratio as the dependent variable and shell TL and time (beginning or end of the experiment) as independent variables. For all regression models reported herein, residual and quantile-quantile plots were examined to ensure homogeneity of variance and normal distribution of data.

We tested whether survival varied as a function of ZnO ENP concentration, mussel size, or their interaction using a two-way ANOVA with mussel size and ZnO ENP concentration as fixed factors. We predicted that survival would decrease with increasing ZnO ENP concentration but that this relationship would be different for small and large mussels causing a significant size x ZnO ENP interaction. We used Tukey's HSD test *post-hoc* to determine which groups significantly differed and Levene's test to ensure homogeneity of variance between groups. R statistical software (The R Foundation for Statistical Computing, version 2.10.1) was used for all analyses.

## Results

### Respiration

**Figure 1:**
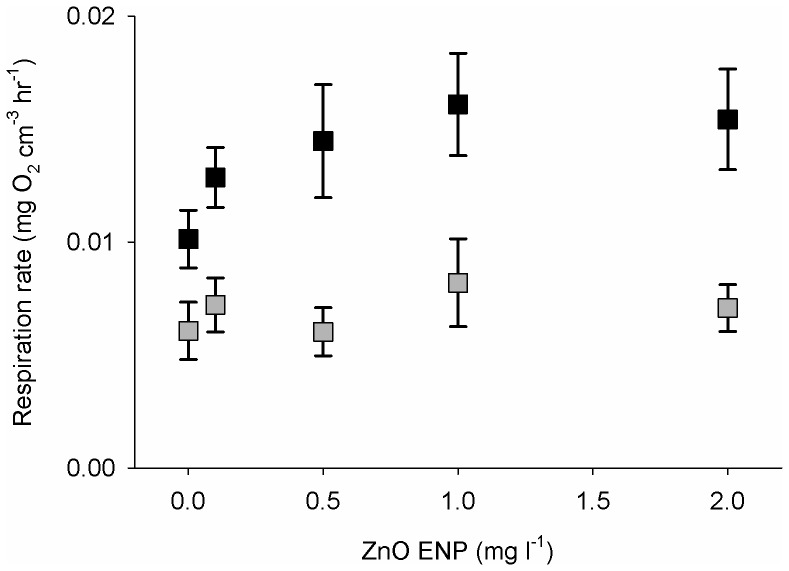
Volume specific respiration rate of mussels exposed to ZnO ENPs. Mean respiration rate ±1 SE of small (black) and large (gray) mussels after 12 wk of exposure to ZnO ENPs. Respiration rate increased with increasing ZnO ENP concentration and was higher for small mussels than large (OLS: *Respiration* = 0.01+2.64×10^−3^ (√*Concentration*)+6.95×10^−3^(Size), r^2^ = 0.24). ZnO ENP concentration was square root transformed to meet the assumptions of OLS regression.

Volume specific respiration rate of mussels was consistently higher for small mussels than large mussels (Figure 1). Respiration rate of mussels significantly varied as a function of ZnO ENP concentration and mussel size (Table 1, OLS: r^2^ = 0.24, *p*<0.0001). Respiration rate of mussels generally increased with increasing ZnO ENP concentration (OLS: t_2, 113_ = 2.27; *p*<0.05) and was significantly higher for small mussels than large (OLS: t_2, 113_ = 5.67, *p*<0.0001).

**Table 1:** Multiple OLS regressions examining effects of ZnO ENP concentration and mussel size on physiological parameters.

	Respiration rate (Parameters×10^3^)	Tissue [Zn]	Zn accumulation rate (small)	Zn accumulation rate (large)	Growth
Intercept	5.02 (1.29)**	138.39 (23.61)**	7.37 (2.90)*	1.00 (1.96)	0.63 (0.06)**
Conc	2.64 (1.17)*	125.58 (18.35)**	0.00 (2.83)	9.46 (1.92)**	−0.19 (0.05)**
Size	6.95 (1.23)**	40.48 (27.12)			0.32 (0.06)**
F	17.77	24.46	<0.01	24.38	21.81
DF	113	36	18	18	326
r^2^	0.24	0.58	<0.01	0.58	0.12

Parameter values reported with standard error in parentheses. For the Zn accumulation rate model, the Concentration x Size interaction term was significant so large and small mussels were run in separate models. ZnO ENP concentration was square root transformed for the respiration rate model.

** *p* value<0.001,

* *p* value<0.05.

### Bioaccumulation

**Figure 2:**
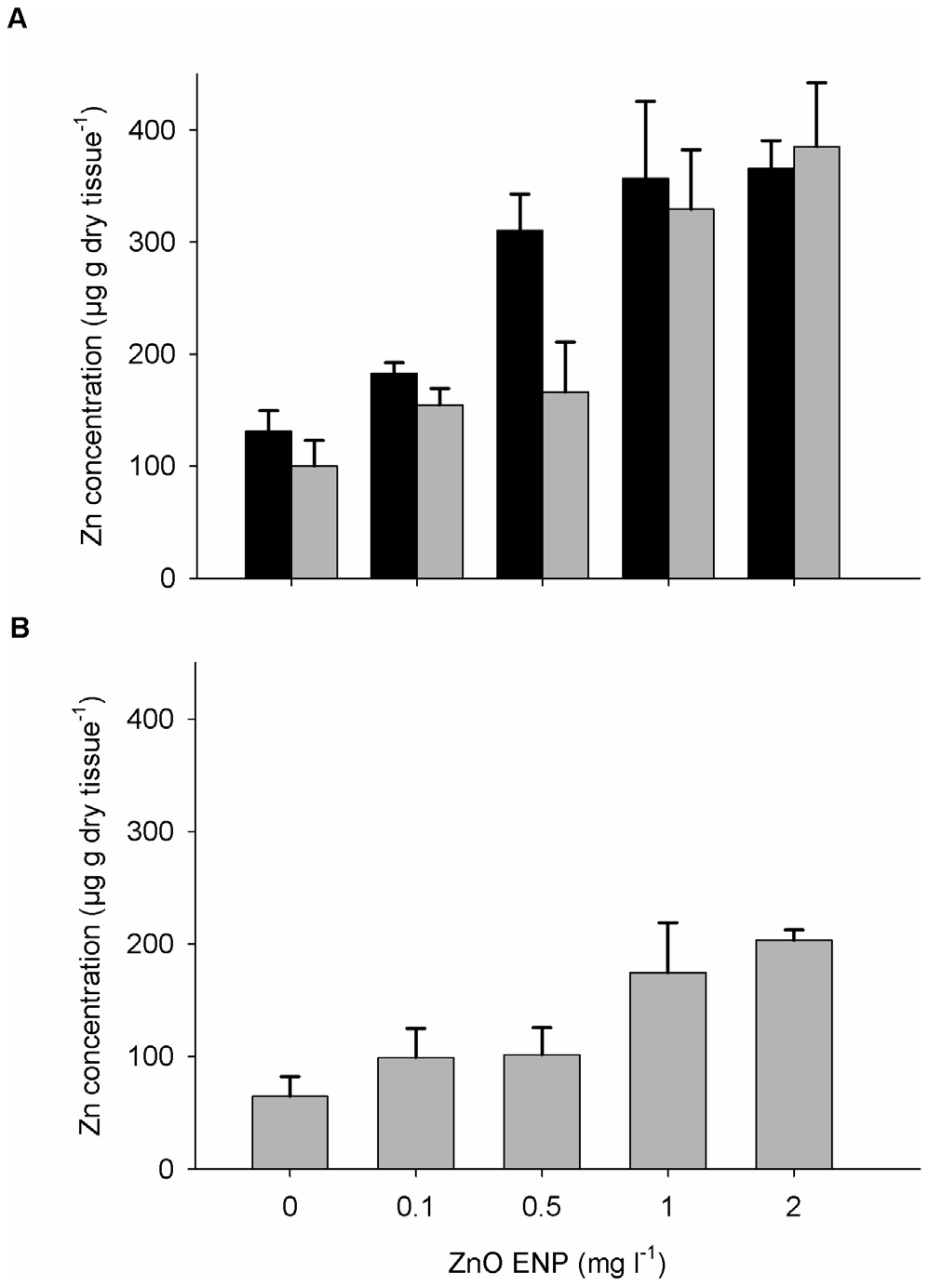
Bioaccumulation of Zn in mussels exposed to ZnO ENPs. Mean Zn concentration per gram dry weight ±1 SE in somatic tissue (A) and gonad (B) of mussels after 12 wk of exposure to ZnO ENPs. Zn concentrations are shown for tissue in small (black) and large (gray) mussels. Zn concentration in somatic tissue of both small and large mussels increased as a function of ZnO ENP concentration (OLS: *Somatic Zn* = 138.39+125.58(*Concentration*)+40.48(*Size*), r^2^ = 0.58). Zn concentration in gonad of large mussels increased as a function of ZnO ENP concentration (OLS: *Gonad Zn* = 81.18+66.88(*Concentration*), r^2^ = 0.51). Zn concentration in gonad from small mussels is not shown due to minimal gonad biomass in this group.

Mussels exposed to ZnO ENPs accumulated Zn in both somatic and reproductive tissues (Figure 2). Zn concentration in somatic tissue increased as a function of ZnO ENP concentration but not mussel size (Table 1, OLS: r^2^ = 0.58; *p*<0.0001). Mussels exposed to 2 mg l^−1^ ZnO ENPs for 12 wk had approximately three fold more Zn in somatic tissue than the control group (Figure 2A). Similarly, Zn concentrations in gonad increased with increasing concentrations of ZnO ENPs (OLS: r^2^ = 0.51; *p*<0.001). Large mussels exposed to 2 mg l^−1^ ZnO ENPs for 12 wk had approximately three times more Zn in gonad than control mussels (Figure 2B).

**Figure 3:**
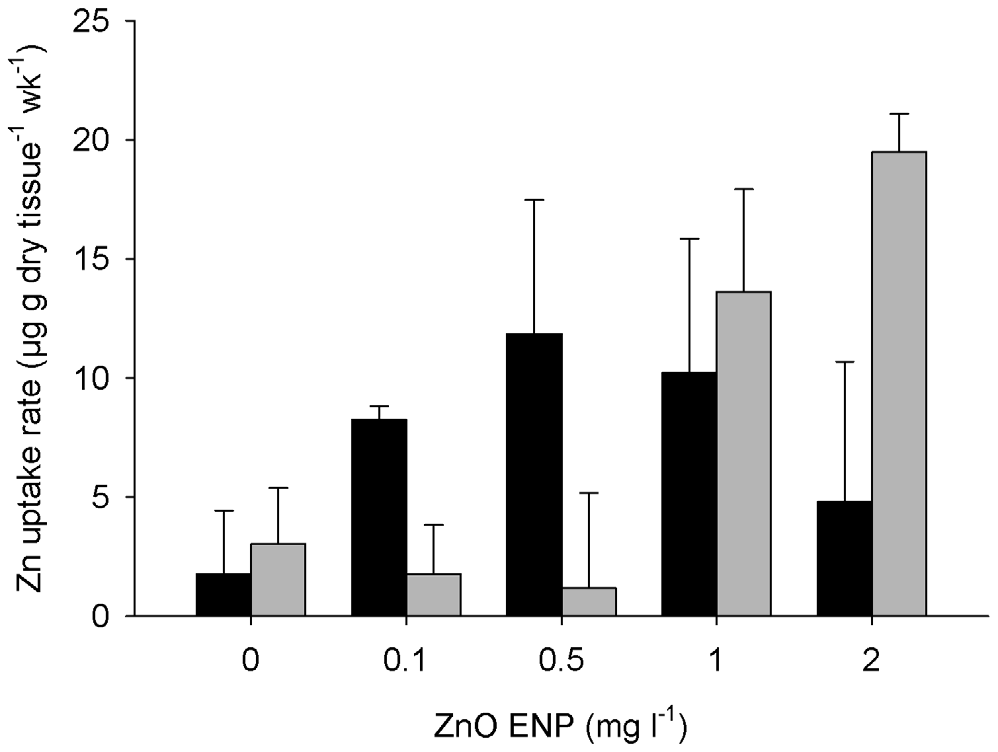
Zn uptake by mussels exposed to ZnO ENPs. Mean rate of Zn uptake by dry weight into somatic tissue ±1 SE for small (black) and large (gray) mussels exposed to ZnO ENPs for 12 wk. Zn accumulation rate did not vary as a function of ZnO ENP concentration in small mussels (OLS: *Zn rate* = 7.37+2.98×10^−3^(*Concentration*), r^2^<0.0001) but increased as a function of ZnO ENP concentration in large mussels (OLS: *Zn rate* = 1.00+9.46(*Concentration*), r^2^ = 0.58).

Rate of Zn accumulation throughout the experiment showed different trends for small mussels than for large mussels (Figure 3). Small mussels accumulated Zn 10 times faster than large mussels at 0.5 mg l^−1^ ZnO ENPs but large mussels accumulated Zn four times faster than small mussels at 2 mg l^−1^. The effect of ZnO ENP concentration on the rate of Zn accumulation significantly varied as a function of mussel size (OLS: t_3, 36_ = 3.42; *p*<0.01), therefore, separate models were run for small and large mussels. For small mussels, the rate of Zn accumulation did not depend on ZnO ENP concentration (Table 1, OLS: r^2^<0.0001, *p*>0.9), but was highly variable for mussels exposed to >0.1 mg l^−1^ ZnO ENPs. For large mussels, Zn accumulation rate increased with increasing concentration of ZnO ENPs (OLS: r^2^ = 0.58, *p*<0.001).

### Growth and survival

**Figure 4:**
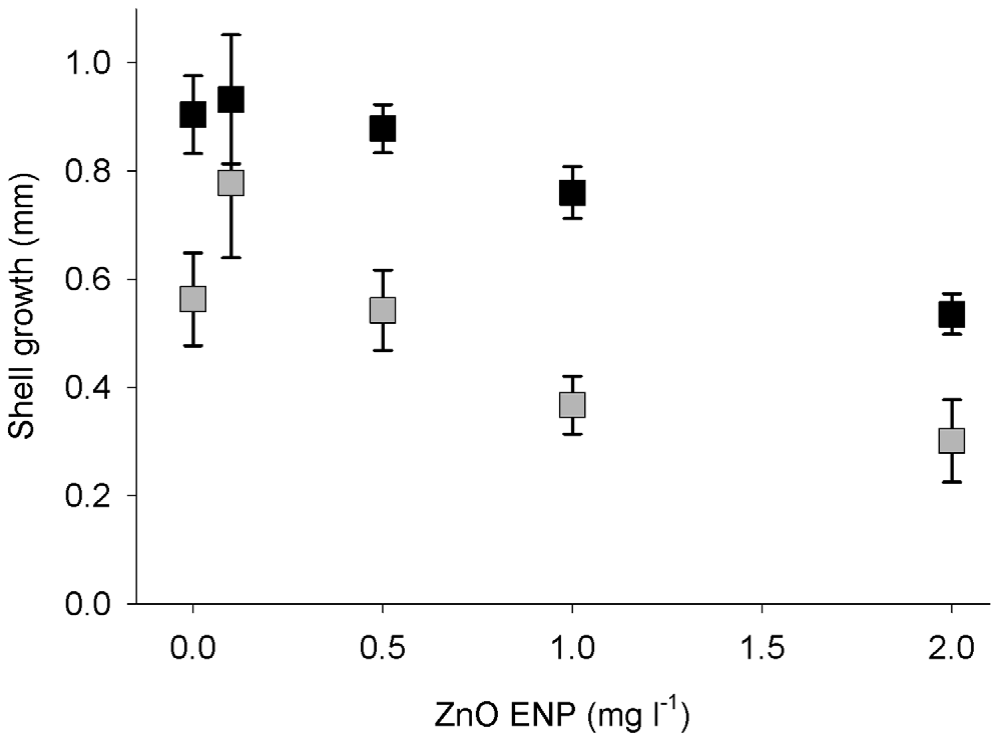
Growth of mussels exposed to ZnO ENPs. Mean growth ±1 SE of small (black) and large (gray) mussels after 12 wk of exposure to ZnO ENPs. ZnO ENPs negatively impacted growth rate for both small and large mussels (OLS: *Growth* = 0.63−0.19(*Concentration*)+0.32(*Size*), r^2^ = 0.12).

The increase in growth, as measured by shell total length (TL), for mussels exposed to ZnO ENPs for 12 wk was less than that of mussels in control groups, although growth was relatively low for all groups (Figure 4). Mussel shell growth significantly varied as a function of ZnO ENP concentration and mussel size (Table 1, OLS: r^2^ = 0.12; *p*<0.0001). Small mussels in the 2 mg l^−1^ exposure group grew 41% less, and large mussels grew 47% less, than the corresponding control group. After 12 wk of exposure to ZnO ENPs, mussel shell growth decreased 0.19 mm for every 1 mg l^−1^ of ZnO ENPs (OLS: t_2, 326_ = −3.87, *p*<0.001). Mean shell growth for small mussels was 20–107% greater than large mussel growth during the experiment. Mussels had lower tissue dry weight to shell TL ratios with increasing shell TL (OLS: t_2, 43_ = −7.43, *p*<0.001). Additionally, at the beginning of the experiment mussels had a lower tissue dry weight to shell TL ratio than mussels at the end of the experiment (OLS: t_2, 43_ = 2.88, *p*<0.01).

**Figure 5:**
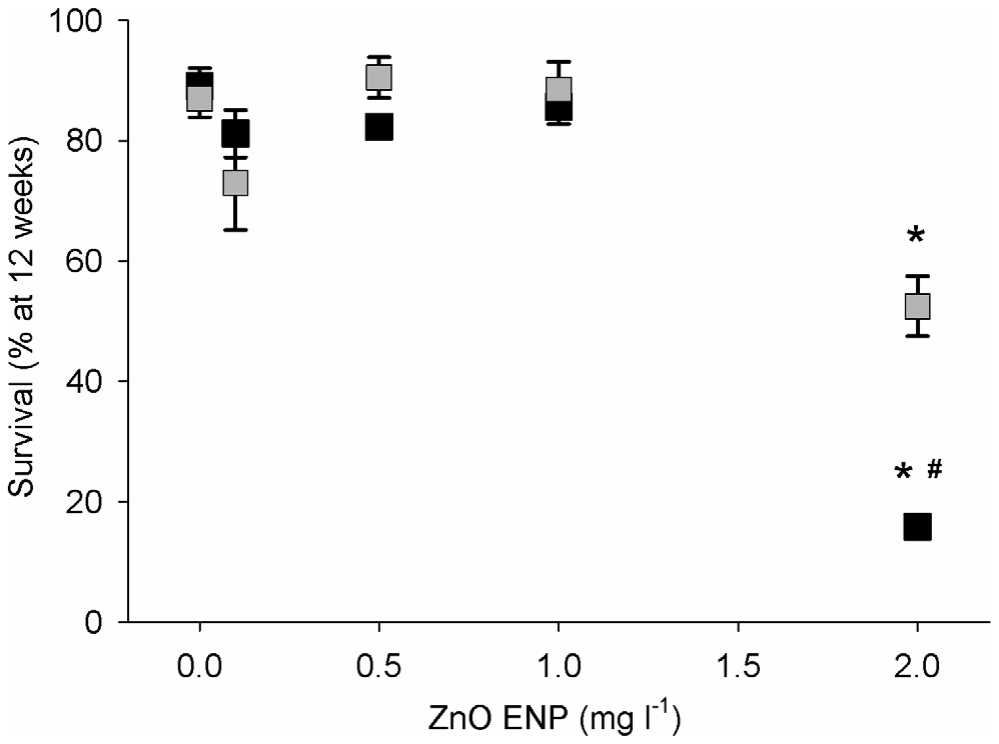
Survival of mussels exposed to ZnO ENPs. Mean survival ±1 SE of small (black) and large (gray) mussels after 12 wk of exposure to ZnO ENPs. The impact of ZnO ENP concentration on survival varied as a function of size (two-way ANOVA: F_4, 30_ = 13.84; *p*<0.0001) but is decreased only in the presence of 2 mg l^−1^ ZnO ENPs and is lower for small mussels than large (Tukey HSD: *p*<0.001). *Indicates significantly lower survival between concentrations within a size group. ^#^Indicates significantly lower survival between sizes within a concentration.

Mean survival of mussels in control groups corrected for sampling was 94% after 6 wk and 91% after 12 wk, and was similar for small and large mussels in all groups except for the highest exposure group (Figure 5). After 6 wk of exposure to 2 mg l^−1^ ZnO ENPs, survival was 91% for large mussels but only 59% for small mussels. After 12 wk of exposure to 2 mg l^−1^ ZnO ENPs, 62% of large mussels survived while only 23% of small mussels survived. The effect of ZnO ENP concentration on survival after 6 wk significantly varied with mussel size (two-way ANOVA: F_4, 30_ = 16.45; *p*<0.0001) and a similar relationship was seen after 12 wk of exposure (two-way ANOVA: F_4, 30_ = 13.84; *p*<0.0001). Small mussels exposed to 2 mg l^−1^ ZnO ENPs had significantly lower survival compared to all other groups after 6 wk of exposure (Tukey HSD: *p*<0.0001). After 12 wk of exposure, small and large mussels exposed to 2 mg l^−1^ ZnO ENPs had significantly lower survival compared to all lower concentrations, and small mussels in this highest exposure group had significantly lower survival compared to the large group at the same exposure concentration (Tukey HSD: *p*<0.001).

## Discussion

We predicted that mussels exposed to ZnO ENPs would increase respiration rates post exposure, increase total tissue Zn, and decrease growth and survival compared with control organisms, and that these impacts would be more pronounced in small mussels. After exposing *M. galloprovincialis* to ZnO ENPs for 12 wk, we observed increases in respiration rates and Zn concentration in tissues. These impacts were related to decreases in growth and survival, and suggest that mussels were expending energy to combat the effects of excess environmental Zn but were unable to meet these demands at our highest exposure concentration, 2 mg l^−1^. Large mussels apparently can tolerate higher concentrations of ZnO ENPs for longer periods of time, as survival was higher for large mussels than that of small mussels after 6 and 12 wk of exposure.

Respiration rates of mussels increased as a function of ZnO ENP concentration after 12 wk of exposure. However, this relationship was more pronounced for small mussels than for large mussels. This difference agreed with our finding of lower survival in the small mussel group exposed to 2 mg l^−1^ ZnO ENPs compared with the large group. We found no statistically significant relationship between respiration rate of mussels as a function of Zn tissue concentrations (results not shown), which is probably the result of high variability of Zn tissue concentrations in mussels in our study, similar to that reported by many others [28]–[30]. Decreased growth rates in the presence of ZnO ENPs combined with increased respiration after exposure suggest that mussels were using energy to detoxify Zn, remove Zn from tissues, or repair damage that resulted from high concentrations of Zn instead of using this energy to produce new tissue or shell.

Shell growth was very low for all mussels in our study (<1 mm in 12 wk), and somatic tissue in relation to shell length decreased during the study, suggesting that mussels were food limited. Assuming each mussel in each tank consumed the same amount of phytoplankton, each mussel in our experiment received between 8×10^5^ and 1×10^6^ cells d^−1^ at the beginning of the experiment and more as the experiment progressed due to mussel mortality and sampling. Previous work suggests that mussels of the size class used in our experiment can ingest as much as 5×10^5^ to 3×10^6^ cells h^−1^ depending on flow rate and phytoplankton concentration in the water [31]. However, food limitation and slow growth is common in natural populations of mussels [32]–[34], unlike the typical laboratory condition of *ad libitum* feeding. Furthermore, ZnO ENPs decrease population growth of marine phytoplankton [8], making it likely that phytoplankton productivity and mussel food supplies will be reduced in zones of ZnO ENP contamination. Taken together, our results suggest that increased ZnO ENP concentrations will increase stress in marine mussels, and that this stress may be further exacerbated under food-limited conditions.

The accumulation of ENPs in bivalves is an environmental concern considering the potential for toxic impacts as well as trophic transfer and even exposure to humans. While research suggests that bivalves accumulate ENPs, the effects of this accumulation are rarely studied. *M. galloprovincialis* accumulate both Ce and Zn when exposed to CeO_2_ and ZnO ENPs but reject large portions of both in their pseudofeces [35]. In marine mesocosms dosed with gold nanorods, clams have been found to be one of the main sinks of gold [36] presumably due to the large amount of water filtered by the clams. Freshwater clams have been shown to uptake and excrete gold ENPs, both aggregated and stabilized, with larger diameter particles being cleared from the water column more quickly than smaller diameter particles [37]. *In vitro* studies of *Mytilus* gill tissue indicated uptake of Fe ENPs but suggest no difference in toxicity between Fe ENPs and dissolved Fe, even though the different forms of Fe followed different uptake routes [38]. Additionally, soluble and insoluble forms of Zn accumulate in the rock oyster, *Saccostrea cucullata*, and are then transferred to the predatory whelk, *Thais clavigera*, with a trophic transfer factor >1, thus indicating the potential for trophic transfer as well as biomagnification [39]. This body of work combined with our results suggest that mussels and other bivalves will accumulate ENPs, but it is unknown if these will remain as ENPs in the organism, be transferred to other organisms that prey on them, or influence organisms differently than the bulk forms of these materials.

As ZnO ENPs dissolve rapidly in seawater [8], the majority of toxicity observed in our study was probably due to Zn^2+^, thus comparisons with Zn toxicity work may prove informative. After a 24 h exposure to Zn and transfer to clean water, *M. edulis* survival decreases for more than 3 wk before leveling off [25]. These researchers report an LC_50_ of 20.8 mg l^−1^, much higher than the concentrations used in our study. However, the same study also indicated effects on byssal thread production with an EC_50_ of 0.64 mg l^−1^ and on shell opening response with an EC_50_ of 1.35 mg l^−1^. Similarly, a study with the freshwater mussel, *Dreissena polymorpha*, found the same EC_50_ value for filtration rate (1.35 mg l^−1^) and a no observed effect concentration of 0.19 mg l^−1^ of Zn [40]. These sublethal impacts closely resemble our experimental findings.

Mussels in our study accumulated Zn to similar concentrations as that reported in the literature. Studies on *M. edulis* show that the majority of uptake occurs within the first few hours of exposure [29]. While exposure concentrations are much higher in many other experiments, resulting Zn accumulation is similar to our findings. Higher concentrations are seen in wild *M. edulis* collected from polluted sites with larger mussels having the highest Zn content by dry weight [24], most likely due to longer exposure times. Zn concentration in gonad was much lower compared to somatic tissue in wild *M. edulis*, and this led Lobel and Wright [28] to assert that Zn does not accumulate in gonad. While we found less Zn in mussel gonad than in somatic tissue, our results indicate increased concentrations of Zn in gonad of mussels exposed to ZnO ENPs compared to controls. These discrepancies illustrate the need for further work in this area to determine whether Zn accumulation patterns are different for nano-particulate forms than dissolved, ionic species.

Our study suggests ZnO ENP concentrations ≤1 mg l^−1^ may impact population size structure, while concentrations >1 mg l^−1^ may further impact size structure by killing smaller mussels but leaving larger ones that can tolerate this pollutant. Both of these findings have ecological implications, considering the impact that mussel size has on predator-prey interactions. For example, *Mytilus* is the preferred prey of the seastar *Pisaster* but this predator usually does not eat relatively large mussels as they are more difficult to open [41]. Our results imply that ZnO ENPs would have a greater effect on survival of small mussels, thus removing potential prey from the ecosystem. However, it takes several years for *Mytilus* to reach a size refuge above which *Pisaster* cannot consume them [41]. The impacts we observed on growth rate in the presence of ZnO ENPs would extend this time, and thereby potentially increase prey availability to the predator. As *Mytilus* is an ecologically important organism in the intertidal, creating habitat and thus influencing biodiversity, the environmental factors that impact their growth and survival may have meaningful effects on marine benthic community composition and structure [42].

A quantitative understanding of the interplay of mechanisms described in the preceding paragraph is essential for determining the implications of our results for population and community ecology. One powerful modeling framework for predicting the population-level impacts of contaminants from results of individual toxicity tests is Dynamic Energy Budget (DEB) modeling [14], [43], [44]. DEB models describe the ingestion and assimilation of food by organisms and how this energy is used for maintenance, growth, and reproduction. Toxicants alter these processes and impact growth and reproduction of individuals. A DEB model [14] was previously used to estimate the population level consequences for mussels exposed to produced (i.e., hydrocarbon and metal contaminated) water from oil production. The data presented in this paper are much more comprehensive than those available for DEB models produced in earlier work, and we have work in progress using DEB modeling to determine long-term ecological implications of the present work.

As new applications for ENPs are developed, the release of large amounts of these particles into environmental systems may become a reality, thus motivating research to assess potential and realized ecological impacts. A logical approach to ecological assessment is to focus on ecologically valuable species, especially ecosystem engineers that substantially influence other species and ecological processes. By chronically exposing mussels to low levels of ZnO ENPs and examining sublethal effects on physiological parameters, impacts on survival, and the effect of body size on these impacts, our data provide a comprehensive understanding of the effects of ZnO ENPs on individual performance of mussels that can be extrapolated to predict effects on mussel populations using DEB modeling, and can be used to inform us of the potential effects of ENPs on communities of coastal marine species.
